# The Widal Test in General Practice

**Published:** 1907-06-08

**Authors:** 


### Clinical Points.

### THE WIDAL TEST IN GENERAL PRACTICE.

The Widal test * is probably the most certain single test we know of for the confirmation of a diagnosis of typhoid fever. It depends upon the fact that, during the course of the illness, the patient develops substances in his blood-serum which have a peculiar action upon any typhoid bacilli which may be mixed with the serum. The nature of this action is uncertain, but its effect is well known; the substances produced in the serum are called agglutinins, and they seem to make the surface of the typhoid bacilli sticky, so that instead of being evenly distributed through the fluid in which they are suspended, they begin to stick together in clumps, or, as it is termed, to agglutinate. When blood is obtained from the patient in the well-known glass pipette, and sent to the laboratory, the serum is separated from the clot and then diluted with normal saline solution. This dilution is readily carried out upon a cover glass. For example, if a mixture is required in which typhoid bacilli are to be suspended in serum diluted 200 times, the method would be as follows: With a platinum-wire loop, one loopful of the serum would be placed upon a cover glass; nine separate loopfuls of solution would be placed in nine separate little heaps upon the same cover glass, and then the whole mixed up together by means of the platinum wire. One loopful of this mixture would then be transferred to a second cover glass, followed by nine loopfuls of solution as before. One loopful of this would be transferred to a third cover glass, and to it would be added one loopful of a broth culture of typhoid bacilli. The result would be a mixture of typhoid bacilli in serum diluted 1 in 200. The cover glass is then inverted over a ring on a slide and the "hanging-drop" preparation watched under the microscope. It is examined at once, to be sure that every thing is all right; and again in half an hour's time. If the bacilli remain as evenly distributed as they were to begin with, the reaction is negative; if, on the other hand, they have run together into masses or clumps, the reaction is positive and the diagnosis of typhoid fever is confirmed unless the patient has had typhoid fever before. A complete positive Widal's test is, therefore, agglutination of the bacilli within half an hour when mixed with serum diluted 1 in 200. As a rule less dilutions of the serum are tested at the same time—for example, 1 in 20 and 1 in 50. If no clumping occurs even in 1 in 20 dilution, the patient almost certainly has no typhoid fever; if the clumping occurs in 1 in 20 and 1 in 50, but not in 1 in 200, the reaction is partial; and in partial cases it is usual to repeat the test in a few days, when, if the condition is typhoid fever, the reaction will usually be found to have become complete. It takes a certain length of time for the patient to develop the agglutinins in his blood; that is to say, the fever must have existed for a certain length of time before the Widal test will be positive. The reaction is seldom complete before the end of the first week, and more often than not there is only a partial reaction at that time, the full agglutinative power of the serum being reached towards the end of the second week.

If the above definition of a positive Widal's test be adhered to, nothing else but typhoid fever, past or present, will give it; so that a positive reaction obtained by a skilled person leaves one with no doubt as to the typhoid fever. Partial reactions, on the other hand, may be obtained in other febrile processes, such as fungating endocarditis, so that when the standard dilution is taken to be 1 in 50, as is the case in some laboratories, there is a certain amount of uncertainty as to the meaning of the result.

The phenomenon of agglutination is not restricted to typhoid fever; the serum of patients with Malta fever will cause clumping of cultures of the micrococcus melitensis; that of patients with specific gastro-enteritis will cause clumping of Gaertner's bacilli; some forms of dysentery lead to the formation of agglutinins for bacillus dysenteriæ; and it is noteworthy that the agglutinins are specific—that is to say, the serum of typhoid fever patients, though clumping bacillus typhosus, will not clump micrococcus melitensis; and so on for each disease and organism.

It has been sometimes thought that the clumping had something to do with the motility of the typhoid bacilli; this is by no means the case; the agglutination depends upon some purely physical cause, independent not only of the motility, but even of the life of the bacilli; in other words, *Widal's test can be performed with dead cultures of bacillus typhosus*; and it can be done in test tubes without any microscope being needed. Widal, in his original method, used test tubes. It is true that the hanging-drop method, with observations under the microscope, is somewhat better and more certain than the test tube method; but it is of considerable value to the general practitioner to know that he can easily perform the Widal test himself, using a dead culture from which there is no danger of inoculating himself or others with typhoid fever. The outfit required can be purchased all ready for use, for a few shillings; it will keep, and enough material for performing 30 Widal tests can be obtained at a time. The outfit contains a sterile suspension of dead typhoid bacilli; a series of four small test tubes marked "50," "100," "200," and "control"; a tube for collecting the blood, another for diluting the serum, a small pipette for measuring it, and a larger pipette for checking off the required quantity of sterile suspension of bacilli. To use the apparatus, the lobe of the patient's ear is pricked in the same manner as for making a blood count; about 10 drops of blood are collected in the blood tube, and the latter is corked. This is all that need be done at

* Called after the name of its discoverer.

the bedside; the doctor can carry the blood away with him, and do the rest of the test at home. When the serum has separated from the clot, which it will have done in an hour or two, a single drop of it is transferred to the diluting tube, and nine drops of normal saline solution are added to it. Normal saline is water to which salt has been added in the proportion of a full drachm of salt to each pint of water. The actual amount of serum put into the diluting tube does not matter provided exactly nine times as much normal saline as serum is used; and to be accurate in the dilution the small pipette is of service. If the diluted serum is cloudy, it will clear if it be allowed to stand for a few minutes. Meanwhile 20 drops (or at least 20 of the same units as those used in measuring off the serum) of the sterile suspension of typhoid bacilli are measured off into each of the special test tubes, and four drops (or units) of the diluted serum are added to the tube marked 50, two drops or units of the diluted serum to the tube marked 100, and one unit to that marked 200. The same number of typhoid bacilli are present in all the tubes; the serum dilution in the first is 1 in 50; in the second, 1 in 100; in the third, 1 in 200; in the fourth there is no serum, so that it can serve as a control, and for purposes of comparison. The tubes are now corked, well shaken, and allowed to stand in not too cold a place. The reaction consists in the appearance of flocculi, which gradually increase in size and settle to the bottom of the tube. If in about six hours the flocculi have formed and begin to settle to the bottom of the 1 in 200 tube, the reaction is strongly positive; if they have done so in the 1 in 50 and 1 in 100 tubes, but not in the 1 in 200, the reaction is partial; if, on the other hand, the fluid in all the tubes remains uniformly cloudy like that in the control tube to which no serum has been added, the reaction is quite negative.

In all probability the busy practitioner will prefer to send the few drops of blood to the laboratory for examination; but in some cases he may be very glad to know that there is an easy way of his doing the test himself.

